# Down-Regulation by Resveratrol of Basic Fibroblast Growth Factor-Stimulated Osteoprotegerin Synthesis through Suppression of Akt in Osteoblasts

**DOI:** 10.3390/ijms151017886

**Published:** 2014-10-06

**Authors:** Gen Kuroyanagi, Takanobu Otsuka, Naohiro Yamamoto, Rie Matsushima-Nishiwaki, Akira Nakakami, Jun Mizutani, Osamu Kozawa, Haruhiko Tokuda

**Affiliations:** 1 Department of Orthopedic Surgery, Nagoya City University Graduate School of Medical Sciences, Nagoya 467-8601, Japan; E-Mails: kokuryugen@yahoo.co.jp (G.K.); pharma2@gifu-u.ac.jp (T.O.); artemicion2000@gmail.com (N.Y.); yakuri@gifu-u.ac.jp (J.M.); 2 Department of Pharmacology, Gifu University Graduate School of Medicine, Gifu 501-1194, Japan; E-Mails: riemn@gifu-u.ac.jp (R.M.-N.); pharma1@gifu-u.ac.jp (A.N.); okozawa@gifu-u.ac.jp (O.K.); 3 Department of Clinical Laboratory, National Center for Geriatrics and Gerontology, Obu, Aichi 474-8511, Japan

**Keywords:** resveratrol, fibroblast growth factor (FGF-2), osteoprotegerin, osteoblast

## Abstract

It is firmly established that resveratrol, a natural food compound abundantly found in grape skins and red wine, has beneficial properties for human health. In the present study, we investigated the effect of basic fibroblast growth factor (FGF-2) on osteoprotegerin (OPG) synthesis in osteoblast-like MC3T3-E1 cells and whether resveratrol affects the OPG synthesis. FGF-2 stimulated both the OPG release and the expression of OPG mRNA. Resveratrol significantly suppressed the FGF-2-stimulated OPG release and the mRNA levels of OPG. SRT1720, an activator of SIRT1, reduced the FGF-2-induced OPG release and the OPG mRNA expression. PD98059, an inhibitor of upstream kinase activating p44/p42 mitogen-activated protein (MAP) kinase, had little effect on the FGF-2-stimulated OPG release. On the other hand, SB203580, an inhibitor of p38 MAP kinase, SP600125, an inhibitor of stress-activated protein kinase/c-*Jun N*-terminal kinase (SAPK/JNK), and Akt inhibitor suppressed the OPG release induced by FGF-2. Resveratrol failed to affect the FGF-2-induced phosphorylation of p44/p42 MAP kinase, p38 MAP kinase or SAPK/JNK. The phosphorylation of Akt induced by FGF-2 was significantly suppressed by resveratrol or SRT1720. These findings strongly suggest that resveratrol down-regulates FGF-2-stimulated OPG synthesis through the suppression of the Akt pathway in osteoblasts and that the inhibitory effect of resveratrol is mediated at least in part by SIRT1 activation.

## 1. Introduction

Bone metabolism is a sophisticated process composed of osteoblastic bone formation and osteoclastic bone resorption [1]. The resorption of preexisting bone by osteoclasts and the formation of new bone by osteoblasts are strictly coordinated to maintain adequate bone mass and strength. Disordered bone remodeling causes metabolic bone diseases, including osteoporosis and fracture healing distress. In the process of bone remodeling, it is generally recognized that a variety of humoral factors, such as prostaglandins and cytokines, play important roles [2]. Osteoprotegerin, an essential secreted protein in bone turnover, which has inhibitory effects on osteoclast activation, is a member of the tumor necrosis factor receptor family, along with receptor activator of nuclear factor-κB (RANK) [3]. Osteoprotegerin is known to be synthesized in osteoblasts and secreted, bind to RANK ligand (RANKL) as a decoy receptor and prevent RANKL from binding to RANK, resulting in suppression of bone resorption [3]. It has been shown that osteoprotegerin-knock out mice suffer from severe osteoporosis, suggesting that osteoprotegerin is a key regulator of osteoclastogenesis and bone metabolism [4]. It is currently recognized that the RANK/RANKL/osteoprotegerin axis is a major regulatory system for osteoclast formation and action [5].

Basic fibroblast growth factor (FGF-2), one of heparin-binding growth factors, is synthesized by osteoblasts and embedded in bone matrix [6,7]. It has been reported that FGF-2 has a potent stimulatory effect on bone formation [8]. During fracture repair, up-regulation of FGF-2 expression in osteoblasts is detected [9]. Therefore, it is currently recognized that FGF-2 plays a crucial role in fracture healing, bone remodeling and osteogenesis [10]. Regarding the intracellular signaling mechanism of FGF-2 in osteoblasts, we have previously demonstrated that FGF-2 stimulates the synthesis of vascular endothelial growth factor (VEGF), a specific growth factor for endothelial proliferation, through p44/p42 mitogen-activated protein (MAP) kinase and stress-activated protein kinase/c-*Jun N*-terminal kinase (SAPK/JNK) in osteoblast-like MC3T3-E1 cells [11,12]. We have also shown that FGF-2 stimulates interleukin-6 (IL-6) synthesis through p38 MAP kinase in these cells [13]. On the other hand, Akt, also called protein kinase B, has been identified as a downstream target of phosphatidylinositol 3-kinase in a variety of cells, including osteoblasts [14,15,16,17]. We have previously reported that Akt activated by FGF-2 negatively regulates the FGF-2-induced VEGF release in MC3T3-E1 cells [18]. Thus, three MAP kinases and Akt are considered to play important roles cooperatively in the FGF-2-intracellular signaling in osteoblast functions.

Polyphenolic compounds in foods, including vegetables and fruits, have beneficial properties for human beings. It is generally known that the natural food compounds possess antioxidative, anti-inflammatory and antitumor effects on various tissues and cells [19,20]. Among them, resveratrol, a polyphenol found abundantly in red grape and berries, can delay the aging process, extend lifespan and reduce the risk of numerous degenerative diseases [21,22]. The French population reportedly tends to smoke and to take saturated fatty acid in meals, but yet maintain relatively low levels of cardiovascular events, which is due to the many amounts of consumption of wine containing abundant resveratrol [23]. Regarding the health of bone, it has recently been reported that women, who preferentially consume wine, have a lower risk of hip fracture compared to non-drinkers, past drinkers and those with other alcohol preferences [24]. As for the molecular mechanism behind the effect of resveratrol, it has been demonstrated that resveratrol exerts its effects through SIRT1, which is consistent with improved cellular function and organismal health by binding to and enhancing the activity of the nicotinamide adenine dinucleotide (NAD^+^)-dependent deacetylase [25]. NAD^+^ is biosynthesized in the human body as a precursor in nicotinamide and has a role important for energy acquisition as a coenzyme of oxidoreductase. However, the detailed actions of resveratrol in bone metabolism and the exact mechanism have not yet been clarified.

In the present study, we investigated the effect of FGF-2 on osteoprotegerin synthesis and whether resveratrol affects the osteoprotegerin synthesis in osteoblast-like MC3T3-E1 cells. We herein demonstrate that resveratrol suppresses FGF-2-stimulated osteoprotegerin synthesis through the down-regulation of the Akt pathway in MC3T3-E1 cells and that the effect of resveratrol is mediated at least in part via SIRT1 activation.

## 2. Results and Discussion

### 2.1. Results

#### 2.1.1. Effect of Resveratrol on the Fibroblast Growth Factor (FGF-2)-Stimulated Osteoprotegerin Release in MC3T3-E1 Cells

**Figure 1:**
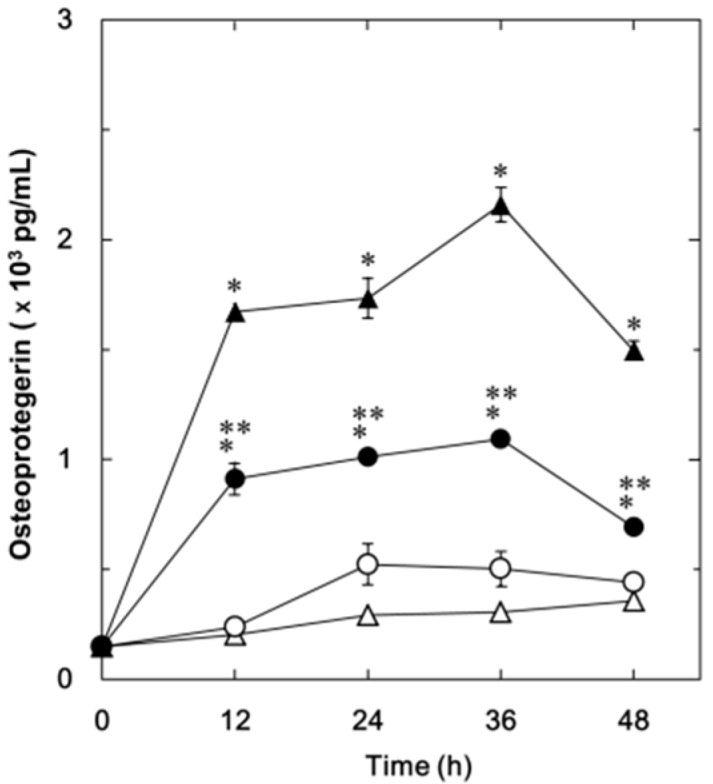
The effect of resveratrol on the FGF-2-stimulated osteoprotegerin release in MC3T3-E1 cells. The cultured cells were pretreated with 50 μM of resveratrol (●,○) or vehicle (▲,△) for 60 min and then stimulated by 30 ng/mL of FGF-2 (●,▲) or vehicle (○,△) for the indicated periods. Osteoprotegerin concentrations of the culture medium were determined by enzyme-linked immunosorbent assay (ELISA). Each value represents the mean ± SEM of triplicate determinations from three independent cell preparations. * *p* < 0.05, compared to the value of control. ** *p* < 0.05, compared to the value of FGF-2 alone.

**Figure 2:**
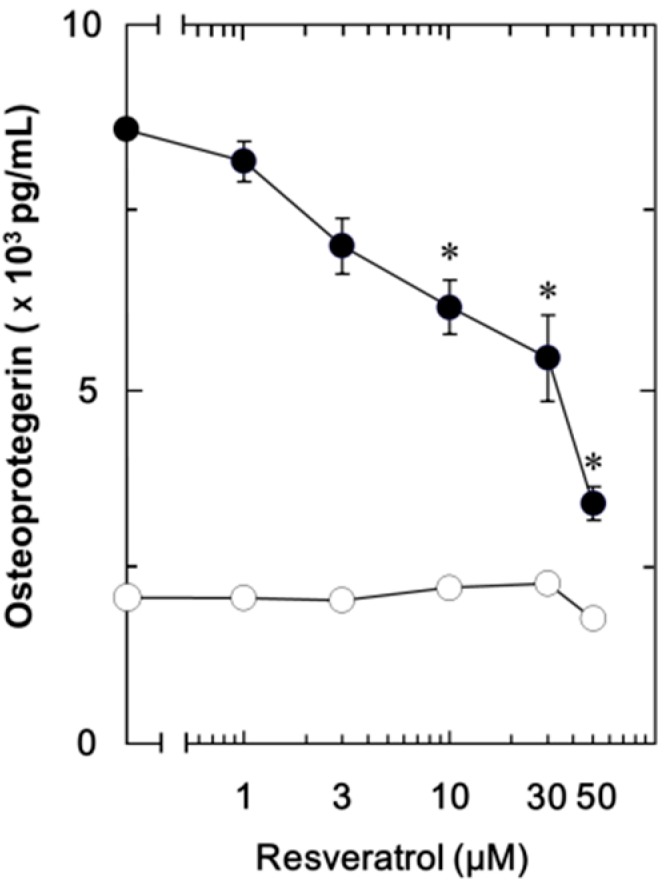
The dose-dependent effect of resveratrol on the FGF-2-stimulated osteoprotegerin release in MC3T3-E1 cells. The cultured cells were pretreated with various doses of resveratrol for 60 min and then stimulated by 30 ng/mL of FGF-2 (●) or vehicle (○) for 48 h. Osteoprotegerin concentrations of the culture medium were determined by ELISA. Each value represents the mean ± SEM of triplicate determinations from three independent cell preparations. * *p* < 0.05, compared to the value of FGF-2 alone.

We have previously demonstrated that FGF-2 stimulates the synthesis of IL-6 and VEGF in osteoblast-like MC3T3-E1 cells [11,12,13]. In the present study, we first investigated whether FGF-2 could stimulate osteoprotegerin synthesis or not in these cells. FGF-2 significantly stimulated the osteoprotegerin release in a time-dependent manner up to 36 h (Figure 1). The maximum effect of FGF-2 on osteoprotegerin release was observed at 36 h and decreased thereafter. We next examined the effect of resveratrol on the FGF-2-stimulated osteoprotegerin release. Resveratrol significantly suppressed the FGF-2-stimulated osteoprotegerin release (Figure 1). The maximum inhibitory effect of resveratrol on the FGF-2-induced osteoprotegerin release was observed at 48 h. The inhibitory effect of resveratrol was dose-dependent within a range between 1 and 50 µM (Figure 2). The effect of resveratrol at 50 μM caused an approximately 75% decrease compared to the osteoprotegerin levels with FGF-2 alone.

#### 2.1.2. Effect of SRT1720 on the FGF-2-Stimulated Osteoprotegerin Release in MC3T3-E1 Cells

SRT1720 is known as a specific and potent synthetic activator of SIRT1 [26]. We investigated the effect of SRT1720 on the FGF-2-stimulated osteoprotegerin synthesis in MC3T3-E1 cells. SRT1720 significantly suppressed the FGF-2-stimulated osteoprotegerin release (Table 1). Treatment with SRT1720 (10 μM) caused an approximately 30% decrease in osteoprotegerin release compared to the levels with FGF-2 alone.

**Table 1:** The effect of SRT1720 on the FGF-2-stimulated osteoprotegerin release in MC3T3-E1 cells. The cultured cells were pretreated with 10 µM of SRT1720 or vehicle for 60 min and then stimulated by 30 ng/mL of FGF-2 or vehicle for 48 h. Osteoprotegerin concentrations of the culture medium were determined by ELISA. Each value represents the mean ± SEM of triplicate determinations from three independent cell preparations. * *p* < 0.05, compared to the value of control. ** *p* < 0.05, compared to the value of FGF-2 alone.

SRT1720	FGF-2	Osteoprotegerin (pg/mL)
−	−	575 ± 9
−	+	3,590 ± 299 *
+	−	514 ± 38
+	+	2,665 ± 72 **

#### 2.1.3. Effects of Resveratrol or SRT1720 on the FGF-2-Induced Expression of mRNA Osteoprotegerin in MC3T3-E1 Cells

**Figure 3:**
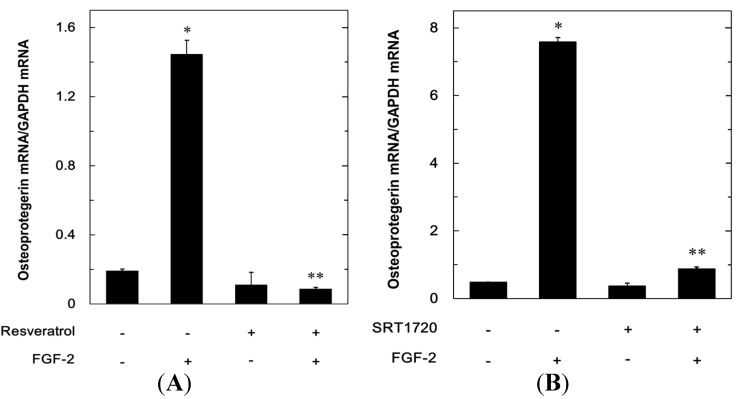
The effects of resveratrol or SRT1720 on the FGF-2-induced expression of osteoprotegerin mRNA in MC3T3-E1 cells. (**A**) The cultured cells were pretreated with 50 µM of resveratrol for 60 min or vehicle, and then stimulated by 30 ng/mL of FGF-2 or vehicle for 3 h; and (**B**) The cultured cells were pretreated with 10 µM of SRT1720 or vehicle for 60 min and then stimulated by 30 ng/mL of FGF-2 or vehicle for 6 h. The expression of osteoprotegerin mRNA and GAPDH mRNA was quantified by real-time RT-PCR. The osteoprotegerin mRNA levels were normalized to those of GAPDH mRNA. Each value represents the mean ± SEM of triplicate determinations from three independent cell preparations. * *p* < 0.05 compared to the value of control. ** *p* < 0.05 compared to the value of FGF-2 alone.

In order to elucidate whether the inhibitory effects of resveratrol or SRT1720 on the FGF-2-stimulated osteoprotegerin release were mediated through a transcriptional event, we examined the effect of resveratrol or SRT1720 on the FGF-2-induced osteoprotegerin mRNA expression by real-time RT-PCR. Resveratrol significantly suppressed the osteoprotegerin mRNA expression levels induced by FGF-2 (Figure 3A). In addition, SRT1720 markedly attenuated the FGF-2-induced osteoprotegerin mRNA expression (Figure 3B).

#### 2.1.4. Effects of PD98059, SB203580, SP600125 or Akt Inhibitor on the FGF-2-Stimulated Osteoprotegerin Release in MC3T3-E1 Cells

In our previous studies [11,12,13], we have shown that FGF-2 stimulates the activation of the major three MAP kinases, p44/p42 MAP kinase, p38 MAP kinase and SAPK/JNK, in osteoblast-like MC3T3-E1 cells. In addition, we recently reported that the FGF-2 stimulates the synthesis of VEGF through the Akt pathway in these cells [18]. Therefore, to investigate whether p44/p42 MAP kinase, p38 MAP kinase, SAPK/JNK or Akt are implicated in the FGF-2-induced osteoprotegerin synthesis in MC3T3-E1 cells, we examined the effects of PD98059, a specific inhibitor of the upstream kinase that activates p44/p42 MAP kinase [27], SB203580, a specific inhibitor of p38 MAP kinase [28], SP600125, a specific inhibitor of SAPK/JNK [29], or Akt inhibitor (1l-6-hydroxymethyl-chiro-inositol 2-(*R*)-2-*O*-*methyl*-3-*O*-octadecylcarbonate) [30] on the osteoprotegerin release stimulated by FGF-2. PD98059 failed to suppress the osteoprotegerin release with or without FGF-2. On the contrary, SB203580, SP600125 or Akt inhibitor significantly decreased the FGF-2-stimulated osteoprotegerin release, suggesting that p38 MAP kinase, SAPK/JNK and Akt are involved in the osteoprotegerin synthesis in MC3T3-E1 cells (Table 2).

**Table 2:** The effects of PD98059, SB203580, SP600125 or the Akt inhibitor on the FGF-2-stimulated osteoprotegerin release in MC3T3-E1 cells. The cultured cells were pretreated with 50 μM of PD98059, 30 μM of SB203580, 10 μM of SP600125, 30 μM of Akt inhibitor or vehicle for 60 min and then stimulated by 30 ng/mL of FGF-2 or vehicle for 48 h. Osteoprotegerin concentrations of the culture medium were determined by ELISA. Each value represents the mean ± SEM of triplicate determinations from three independent cell preparations. * *p* < 0.05, compared to the value of control. ** *p* < 0.05, compared to the value of FGF-2 alone.

Inhibitors	FGF-2	Osteoprotegerin (pg/mL)
−	−	1,052 ± 52
−	+	2,143 ± 80 *
PD98059	−	1,088 ± 48
PD98059	+	2,791 ± 104
SB203580	−	609 ± 29
SB203580	+	1,005 ± 13 **
SP600125	−	879 ± 28
SP600125	+	986 ± 64 **
Akt inhibitor	−	693 ± 28
Akt inhibitor	+	986 ± 64 **

#### 2.1.5. Effects of Resveratrol on the FGF-2-Induced Phosphorylation of p44/p42 MAP Kinase, p38 MAP Kinase or SAPK/JNK in MC3T3-E1 Cells

**Figure 4:**
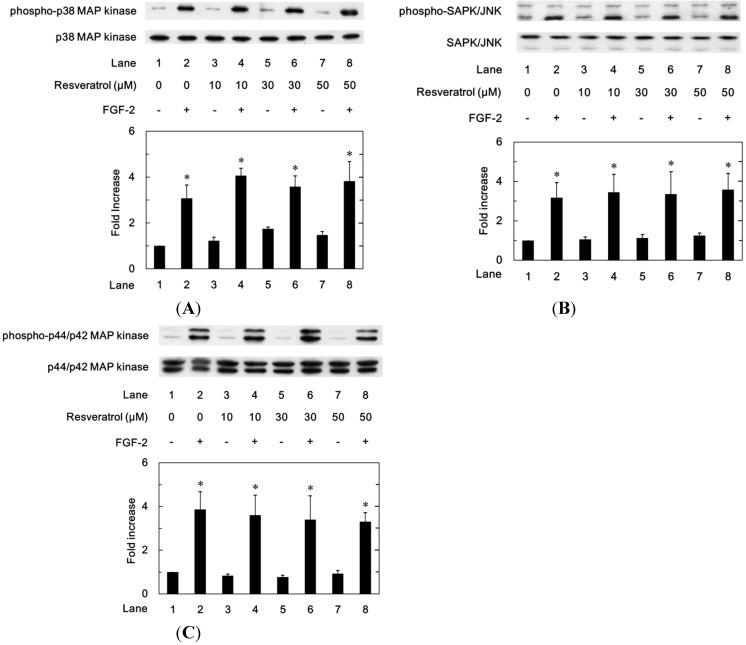
The effects of resveratrol on the FGF-2-induced phosphorylation of p38 MAP kinase (**A**), SAPK/JNK (**B**) or p44/p42 MAP kinase (**C**) in MC3T3-E1 cells. The cultured cells were pretreated with various doses of resveratrol for 60 min and then stimulated by 30 ng/mL of FGF-2 or vehicle for 10 min (**A**) or 20 min (**B**,**C**). The cell extracts were then subjected to SDS-PAGE with subsequent western blot analysis with antibodies against phospho-specific p38 MAP kinase, p38 MAP kinase, phospho-specific SAPK/JNK, SAPK/JNK, phospho-specific p44/p42 MAP kinase or p44/p42 MAP kinase. The histogram shows a quantitative representation of the levels of FGF-2-induced phosphorylation obtained from a laser densitometric analysis of three independent experiments. Each value represents the mean ± SEM of triplicate determinations. * *p* < 0.05, compared to the value of control.

In order to clarify whether resveratrol could modulate the activation of p38 MAP kinase or SAPK/JNK stimulated by FGF-2 or not, we examined the effect of resveratrol on the FGF-2-induced phosphorylation of p38 MAP kinase or SAPK/JNK in MC3T3-E1 cells. However, resveratrol failed to attenuate the FGF-2-induced phosphorylation of p38 MAP kinase (Figure 4A) or SAPK/JNK (Figure 4B). Additionally, we found that resveratrol did not affect the p44/p42 MAP kinase phosphorylation stimulated by FGF-2 (Figure 4C).

#### 2.1.6. Effects of Resveratrol or SRT1720 on the FGF-2-Induced Phosphorylation of Akt in MC3T3-E1 Cells

**Figure 5:**
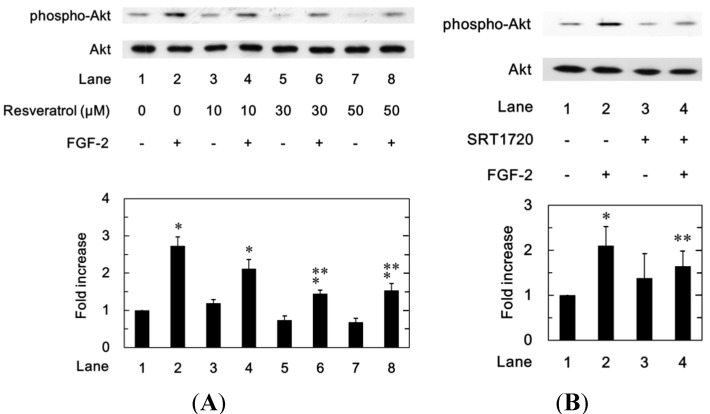
The effects of resveratrol (**A**) or SRT1720 (**B**) on the FGF-2-induced phosphorylation of Akt in MC3T3-E1 cells. The cultured cells were pretreated with various doses of resveratrol (**A**), 10 μM of SRT1720 (**B**) or vehicle for 60 min and then stimulated by 30 ng/mL of FGF-2 or vehicle for 5 min. The cell extracts were then subjected to SDS-PAGE with subsequent western blot analysis with antibodies against phospho-specific Akt or Akt. The histogram shows quantitative representation of the levels of FGF-2-induced phosphorylation obtained from a laser densitometric analysis of three independent experiments. Each value represents the mean ± SEM of triplicate determinations. * *p* < 0.05, compared to the value of control. ** *p* < 0.05, compared to the value of FGF-2 alone.

Furthermore, we next investigated the effect of resveratrol on the phosphorylation of Akt induced by FGF-2. Resveratrol significantly attenuated the FGF-2-induced phosphorylation of Akt in a dose-dependent manner in the range between 10 and 50 µM in MC3T3-E1 cells (Figure 5A). In addition, we examined the effect of SRT1720 on the FGF-2-induced phosphorylation of Akt. SRT1720 significantly suppressed the FGF-2-induced phosphorylation of Akt (Figure 5B). SRT1720 mimicked the suppressive effect of resveratrol on the FGF-2-induced phosphorylation of Akt.

### 2.2. Discussion

In the present study, we showed that resveratrol significantly reduced the FGF-2-stimulated osteoprotegerin release in osteoblast-like MC3T3-E1 cells. In addition, we demonstrated that FGF-2 increased the levels of osteoprotegerin mRNA, and resveratrol reduced the mRNA expression levels of osteoprotegerin up-regulated by FGF-2. Taking our findings into account, it is most likely that the inhibitory effect of resveratrol on FGF-2-stimulated osteoprotegerin release were mediated through a transcriptional event. In co-cultures of spleen cells and osteoblasts, it has been reported that FGF-2 suppresses osteoprotegerin production [31]. The discrepancy may be due to the differences of experimental conditions. Therefore, we further investigated the exact mechanism behind the resveratrol-effects on the FGF-2-stimulated osteoprotegerin synthesis in osteoblasts. It has been proposed that the effects of resveratrol are exerted through SIRT1 activation [32]. SIRT1 is an essential modulator of pathways downstream of caloric restriction that has beneficial effects on glucose homeostasis and insulin sensitivity [33]. SRT1720 is known to be a synthetic compound that has been identified for its ability to activate SIRT1 [26]. We showed that SRT1720 suppressed the FGF-2-induced osteoprotegerin release and expression of osteoprotegerin mRNA, as well as resveratrol in MC3T3-E1 cells. Thus, our findings suggest that the inhibitory effect of resveratrol on the FGF-2-stimulated osteoprotegerin synthesis is mediated at least in part by the activation of SIRT1 in osteoblast-like MC3T3-E1 cells.

**Figure 6:**
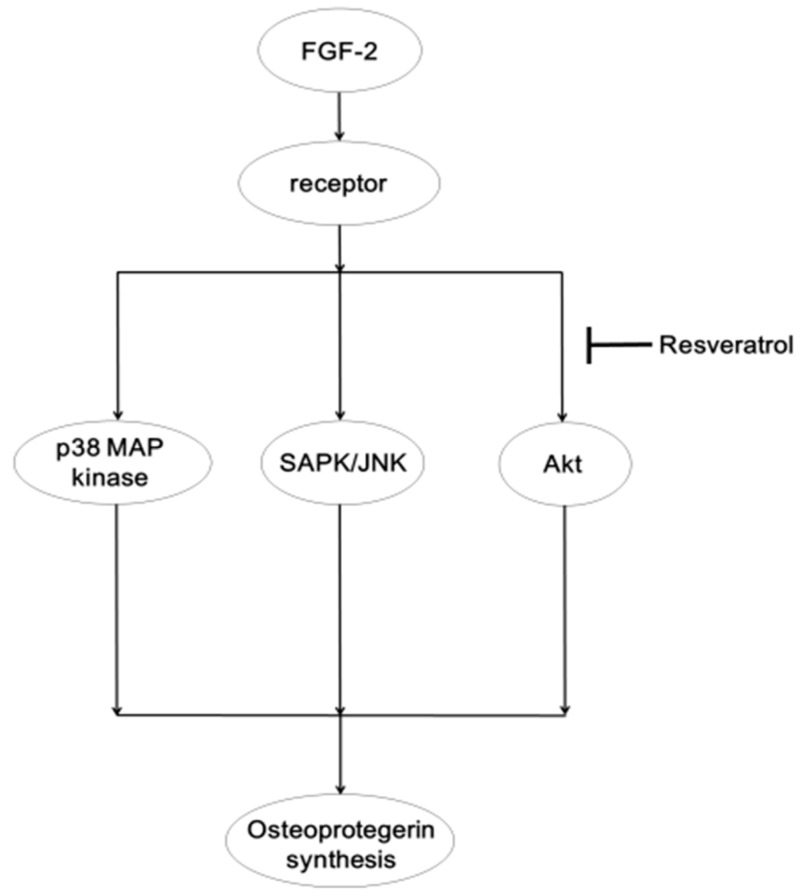
Schematic illustration of the regulatory mechanism of resveratrol on the FGF-2-induced osteoprotegerin synthesis in osteoblast-like MC3T3-E1 cells. Resveratrol reduces FGF-2-induced osteoprotegerin synthesis by inhibiting the Akt pathway without affecting p38 MAP kinase or SAPK/JNK and functions at a point upstream of Akt.

The MAP kinase superfamily plays an essential role in a variety of cellular functions, including proliferation, differentiation and survival [34]. Among the MAP kinase superfamily, it is generally known that three MAP kinases, p44/p42 MAP kinase, p38 MAP kinase and SAPK/JNK, are central elements used by mammalian cells to transduce the diverse messages [35]. As for the intracellular signaling system of FGF-2 in osteoblasts, we have previously demonstrated that p44/p42 MAP kinase, p38 MAP kinase and SAPK/JNK are activated by FGF-2 in osteoblast-like MC3T3-E1 cells [11,12,13]. Additionally, we have reported that the Akt pathway plays a negative role in the FGF-2-stimulated VEGF release in these cells [18]. Therefore, we examined the effects of PD98059 [27], SB203580 [28], SP600125 [29] or Akt inhibitor [30] on the osteoprotegerin release induced by FGF-2 and showed that SB203580, SP600125 or the Akt inhibitor, but not PD98059, significantly reduced the FGF-2-stimulated osteoprotegerin release in MC3T3-E1 cells. Therefore, it is probable that p38 MAP kinase, SAPK/JNK and Akt act as positive regulators in the FGF-2-stimulated osteoprotegerin synthesis in osteoblast-like MC3T3-E1 cells. In addition, we showed that resveratrol attenuated the FGF-2-induced phosphorylation of Akt, without affecting the FGF-2-induced phosphorylation of p38 MAP kinase or SAPK/JNK in these cells. We further found that resveratrol had little effect on the FGF-2 induced phosphorylation of p44/p42 MAP kinase. Therefore, these results strongly suggest that resveratrol affects the FGF-2-signaling in osteoprotegerin synthesis at a point upstream of Akt in osteoblast-like MC3T3-E1 cells. In these cells, we have recently shown that resveratrol down-regulates prostaglandin D_2_-stimulated osteoprotegerin synthesis through inhibiting p38 MAP kinase and SAPK/JNK [36] and that the osteoprotegerin synthesis induced by prostaglandin F_2__α_ is attenuated by resveratrol through the suppression of p44/p42 MAP kinase, p38 MAP kinase and SAPK/JNK [37]. It is generally recognized that prostaglandin receptors belong to seven-transmembrane helical receptors, also known as G protein-coupled receptors, whereas FGF-2 receptors are tyrosine kinase-including single-span transmembrane receptors [38,39]. Therefore, it seems likely that the discrepancies between the present findings and these previous studies are due to the difference of receptor structure and the following signaling system. Furthermore, we demonstrated that SRT1720 reduced the FGF-2-induced phosphorylation of Akt, as well as resveratrol. Based on our findings as a whole, it is most likely that resveratrol suppresses the FGF-2-stimulated osteoprotegerin synthesis, at least in part by activation of SIRT1 in osteoblast-like MC3T3-E1 cells, and that the inhibitory effect of resveratrol is exerted at a point upstream of Akt. With regard to the mechanism of the SIRT1 effects, it has been reported that SIRT1-mediated deacetylation of Akt regulates its binding to phosphatidylinositol (3,4,5)-triphosphate and its activation [40]. Thus, it is possible that SIRT1 regulates Akt activity through the deacetylation in osteoblast-like MC3T3-E1 cells. The potential mechanism of resveratrol in FGF-2-stimulated osteoprotegerin synthesis in osteoblasts is summarized in Figure 6.

FGF-2 is synthesized by osteoblasts and embedded into bone matrix in the process of bone remodeling [10]. During bone resorption by osteoclasts, the embedded FGF-2 is released and affects osteoblast precursors as a local osteogenic factor. The potent anabolic effects of FGF-2 on osteogenesis and bone fracture repair are firmly established [6,10]. On the other hand, osteoprotegerin, which has been identified as an osteoclastogenesis inhibitory factor, is known to function in the regulation of RANKL-mediated osteoclastic bone resorption, the initial step of bone remodeling [1]. To maintain the quality and quantity of bone, proper bone remodeling is required to remove old fragile skeleton and to renew the construction. Taking these findings into account, our present results showing the inhibitory effect of resveratrol on the FGF-2-stimulated osteoprotegerin synthesis by osteoblasts might provide its new aspect as an up-regulator of bone tissue quality through suppression of excessive bone turn over and the initiation of the adequate bone remodeling process. Further investigation would be necessary to clarify the detailed mechanism behind the effects of resveratrol on bone metabolism.

## 3. Experimental Section

### 3.1. Materials

Recombinant human FGF-2 was obtained from Invitrogen BV., (Breda, Netherlands). Resveratrol, SRT1720, PD98059, SB203580, SP600125 and the Akt inhibitor (1l-6-hydroxymethyl-chiro-inositol 2-(*R*)-2-*O*-*methyl*-3-*O*-octadecylcarbonate) were obtained from Calbiochem-Novabiochem Co. (La Jolla, CA, USA). Mouse osteoprotegerin enzyme-linked immunosorbent assay (ELISA) kits were obtained from R&D Systems, Inc. (Minneapolis, MN, USA). Phospho-specific p44/p42 MAP kinase antibodies, p44/p42 MAP kinase antibodies, phospho-specific p38 MAP kinase antibodies, p38 MAP kinase antibodies, phospho-specific SAPK/JNK antibodies, SAPK/JNK antibodies, phospho-specific Akt antibodies (Thr308) and Akt antibodies were obtained from Cell Signaling Technology, Inc. (Beverly, MA, USA). An ECL western blotting detection system was obtained from GE Healthcare (Buckinghamshire, UK). Other materials and chemicals were obtained from commercial sources. Resveratrol, SRT1720, PD98059, SB203580, SP600125 and the Akt inhibitor were dissolved in dimethyl sulfoxide. The maximum concentration of dimethyl sulfoxide was 0.1%, which did not affect either the assay for osteoprotegerin or the western blot analysis.

### 3.2. Cell Culture

Cloned osteoblast-like MC3T3-E1 cells, which have been derived from newborn mouse calvaria [41], were maintained as previously described [42]. Briefly, the cells were cultured in α-minimum essential medium (α-MEM, SIGMA-ALDRICH, Co., St-Louis, MO, USA) containing 10% fetal bovine serum (FBS, PAA Laboratories GmbH, Pasching, Austria) at 37 °C in a humidified atmosphere of 5% CO_2_/95% air. The cells were seeded into 35 mm-diameter dishes (5 × 10^4^ cells/dish) or 90 mm-diameter dishes (2 × 10^5^ cells/dish) in α-MEM containing 10% FBS. After five days, the medium was exchanged for α-MEM containing 0.3% FBS. The cells were then used for experiments after 48 h.

### 3.3. Assay for Osteoprotegerin

The cultured cells were pretreated with various doses of resveratrol, SRT1720, PD98059, SB203580, SP600125 or the Akt inhibitor for 60 min, then stimulated by 30 ng/mL of FGF-2 or vehicle in 1 mL of α-MEM containing 0.3% FBS and then incubated for the indicated periods. The conditioned medium was collected, and osteoprotegerin concentration in the medium was measured by a mouse osteoprotegerin ELISA kit according to the manufacturer’s protocol.

### 3.4. Real-Time RT-PCR

The cultured cells were pretreated with 50 µM of resveratrol, 10 µM of SRT1720 or vehicle for 60 min and then stimulated by 30 ng/mL of FGF-2 or vehicle in α-MEM containing 0.3% FBS for the indicated periods. Total RNA was isolated and transcribed into complementary DNA using the Trizol reagent (Invitrogen Co., Carlsbad, CA, USA) and the Omniscript Reverse Transcriptase Kit (QIAGEN Inc., Valencia, CA, USA), respectively. Real-time RT-PCR was performed using a Light Cycler system in capillaries with the Fast Start DNA Master SYBR Green I provided with the kit (Roche Diagnostics, Basel, Switzerland). Sense and antisense primers for mouse osteoprotegerin or glyceraldehyde-3-phosphate dehydrogenase (GAPDH) mRNA were purchased from Takara Bio Inc., (Tokyo, Japan) (primer set ID: MA026526). The amplified products were determined by a melting curve analysis and agarose electrophoresis. The osteoprotegerin mRNA levels were normalized to those of GAPDH mRNA.

### 3.5. Western Blot Analysis

The cultured cells were pretreated with various doses of resveratrol or SRT1720 for 60 min and were then stimulated by 30 ng/mL of FGF-2 or vehicle for the indicated periods. When indicated, the cells were washed twice with phosphate-buffered saline and then lysed, homogenized and sonicated in a lysis buffer containing 62.5 mM Tris/HCl, pH 6.8, 2% sodium dodecyl sulfate (SDS), 50 mM dithiothreitol and 10% glycerol. SDS-polyacrylamide gel electrophoresis (PAGE) was performed by the method described by Laemmli [43] in 10% polyacrylamide gels. A western blot analysis was performed as described previously [44] by using phospho-specific p44/p42 MAP kinase antibodies, p44/p42 MAP kinase antibodies, phospho-specific p38 MAP kinase antibodies, p38 MAP kinase antibodies, phospho-specific SAPK/JNK antibodies, SAPK/JNK antibodies, phospho-specific Akt antibodies or Akt antibodies, with peroxidase-labeled antibodies raised in goat against rabbit IgG being used as secondary antibodies. The peroxidase activity on the polyvinylidene difluoride (PVDF) membrane was visualized on X-ray films by means of an Enhanced chemiluminescence (ECL) western blotting detection system.

### 3.6. Densitometric Analysis

A densitometric analysis of the expression was performed using a scanner and an image analysis software program (ImageJ version 1.32, National Institutes of Health, Bethesda, MD, USA). The background-subtracted signal intensity of each phosphorylation signal was normalized to the respective total protein signal and plotted as the fold increase in comparison to control cells without stimulation.

### 3.7. Statistical Analysis

The data were analyzed by an ANOVA, followed by the Bonferroni method for multiple comparisons between pairs, and values of *p* < 0.05 were considered to be statistically significant. All data are presented as the mean ± SEM of triplicate determinations from three independent cell preparations.

## 4. Conclusions

In conclusion, our results strongly suggest that resveratrol suppresses FGF-2-stimulated osteoprotegerin synthesis through the down-regulation of the Akt pathway in osteoblasts and that the suppressing effect of resveratrol is mediated at least in part by SIRT1 activation.
